# Transparent machine learning suggests a key driver in the decision to start insulin therapy in individuals with type 2 diabetes

**DOI:** 10.1111/1753-0407.13361

**Published:** 2023-03-08

**Authors:** Nicoletta Musacchio, Rita Zilich, Paola Ponzani, Giacomo Guaita, Carlo Giorda, Rebeca Heidbreder, Pierluigi Santin, Graziano Di Cianni

**Affiliations:** ^1^ AMD past President ‐ AMD AI National Group Coordinator Milan Italy; ^2^ Mix‐x SRL Ivrea Italy; ^3^ Diabetes and Endocrinology Unit Local Health Autlhority 4 Chiavari Chiavari Italy; ^4^ Diabetes and Endocrinology Unit ASL SULCIS Iglesias Italy; ^5^ Diabetes and Endocrinology Unit ASL TO5 Chieri Italy; ^6^ PsychResearchCenter, LLC Powhatan Virginia USA; ^7^ Deimos Udine Italy; ^8^ USL Tuscany Northwest Location Livorno, Diabetes and Metabolic Disease Livorno Italy

**Keywords:** artificial intelligence, insulin therapy, machine learning, therapeutic inertia, type 2 diabetes, 2型糖尿病, 治疗惯性, 机器学习, 人工智能

## Abstract

**Aims:**

The objective of this study is to establish a predictive model using transparent machine learning (ML) to identify any drivers that characterize therapeutic inertia.

**Methods:**

Data in the form of both descriptive and dynamic variables collected from electronic records of 1.5 million patients seen at clinics within the Italian Association of Medical Diabetologists between 2005–2019 were analyzed using logic learning machine (LLM), a “clear box” ML technique. Data were subjected to a first stage of modeling to allow ML to automatically select the most relevant factors related to inertia, and then four further modeling steps individuated key variables that discriminated the presence or absence of inertia.

**Results:**

The LLM model revealed a key role for average glycated hemoglobin (HbA1c) threshold values correlated with the presence or absence of insulin therapeutic inertia with an accuracy of 0.79. The model indicated that a patient's dynamic rather than static glycemic profile has a greater effect on therapeutic inertia. Specifically, the difference in HbA1c between two consecutive visits, what we call the HbA1c gap, plays a crucial role. Namely, insulin therapeutic inertia is correlated with an HbA1c gap of <6.6 mmol/mol (0.6%), but not with an HbA1c gap of >11 mmol/mol (1.0%).

**Conclusions:**

The results reveal, for the first time, the interrelationship between a patient's glycemic trend defined by sequential HbA1c measurements and timely or delayed initiation of insulin therapy. The results further demonstrate that LLM can provide insight in support of evidence‐based medicine using real world data.

## Graphical abstract

Highlights

ML suggests that when the HbA1c gap from a previous visit is >11 mmol/mol (1.0%) there is a greater probability that insulin therapy will be initiated, but when HbA1c gap is <6.6 mmol/mol (0.6%), a timely initiation of insulin therapy is less probable. Furthermore, for individuals initiated on insulin in a timely manner, the HbA1c gap is systematically higher than for those patients who have experienced clinical inertia. This key driver correlated with insulin therapy initiation could help combat clinical inertia.

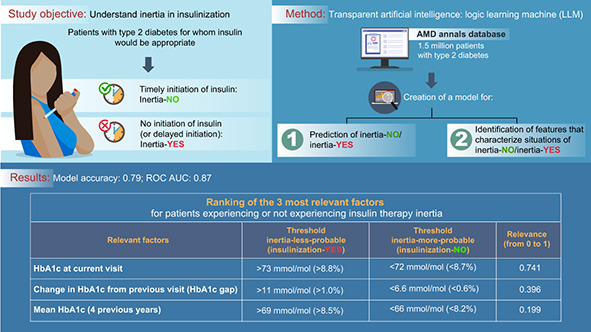

## 摘要

**目的:** 本研究的目的是利用透明机器学习(ML)建立预测模型, 以识别表征治疗惯性的任何驱动因素。

**材料和方法:** 使用逻辑学习机(LLM)(一种“clear box”ML技术)分析了从2005‐2019年意大利医学糖尿病学家协会(AMD)诊所的150万名患者的电子记录中收集的描述性和动态变量形式数据。数据经过第一阶段的建模, 以允许ML自动选择与惯性相关的最相关因素, 然后再经过四个建模步骤, 对关键变量进行个性化, 以区分惯性的存在或不存在。

**结果:** LLM模型揭示了平均HbA_1c_阈值与胰岛素治疗惯性的存在或不存在之间的关键作用, 准确率为0.79。该模型表明, 与静态血糖代谢情况相比, 患者动态血糖代谢情况对治疗惯性具有更大的影响。具体而言, 两次连续访问之间的HbA_1c_差异(我们称之为HbA_1c_间隙)发挥了至关重要的作用。即, 胰岛素治疗惯性与HbA_1c_间隙<6.6mmol/L(0.6%)有关, 但与HbA_1c_间隙>11mmol/L(1.0%)无关。

**结论:** 该结果揭示了由连续HbA_1c_测量确定的患者血糖趋势与及时或延迟开始胰岛素治疗之间的相互关系。研究结果进一步表明, LLM可以利用真实世界的数据为循证医学(EBM)提供支持。

## 1 INTRODUCTION

In spite of abundant evidence demonstrating that in type 2 diabetes early glycemic control is correlated with a reduction in long‐term complications,^1^ data from many health systems indicate that delay in initiation and/or intensification of insulin therapy remains systemic.^2^, ^3^ The end result is that glycemic control in type 2 diabetes is globally inadequate, and individuals live years with poor glycemic control.^4^, ^5^ Several studies have provided explanations for the motivation behind the delay in glycemic control,^6^, ^7^, ^8^ but none, to our knowledge, have examined real world data with artificial intelligence (AI) techniques to identify which factors are more likely to be associated with a provider's behavior (presence or absence of inertia) with regard to initiation of insulin therapy.

In 2005, the Italian Association of Medical Diabetologists (AMD) initiated the Annals project that led to the creation of a network of diabetes clinics, representing half of the total number of diabetes clinics in Italy, with the aim of monitoring, standardizing, and sharing the main parameters used for the evaluation of the quality of care given to patients. In this way, the AMD Annals were able to collect from patient electronic records up to 180 parameters, including clinical, pharmacological, organization related, and provider related, from each patient^9^, ^10^, ^11^ to create “big data” sets (approximately 1.5 million patients and about 9 million visits), which permitted a thorough analysis using AI techniques.

Previously published studies have utilized AI methods to investigate other aspects of diabetes and have yielded promising results.^12^ The present study applied AI techniques, specifically a transparent machine learning (ML) methodology, to overcome the problems associated with “black box” AI algorithms, which can deliver performant models but do not furnish explanations as to how the results were obtained.^13^, ^14^ The transparent ML technique used in this study is based on a proprietary algorithm of “explainable artificial intelligence” known as logic learning machine (LLM), which yields performance that is on par to the best ML algorithms while at the same time allowing full control over the algorithmic logic and permitting the correlation between predictive factors and outcome.^14^ LLM has already been used to great effect in the analysis of biomedical datasets included in the Statlog benchmark.^15^, ^16^ For the purposes of this study, LLM was used to generate predictive and explanatory models that identify the combination of factors (clinical, personal, organizational) correlated with provider inertia in situations which would otherwise require the initiation of insulin therapy. This approach has already been previously used to good effect to analyze other types of clinical data.^17^, ^18^

## 2 MATERIAL AND METHODS

### 2.1 Participants

Data from people with type 2 diabetes were obtained from electronic medical records located on the AMD Annals database. The medical records are from patients who, prior to being referred to one of the clinics within the AMD, showed a high risk for the development of type 2 diabetes or had lab values that were indisputably consistent with type 2 diabetes. No patient referred to one of the diabetes clinics was initiated on insulin prior to their first visit at the clinic. Every patient had visited at least one of the Italian diabetes clinics between 2005 and the first half of 2019.^5^ A total of 2.3 billion data points corresponding to information on 1 186 247 people with a confirmed diagnosis of type 2 diabetes (as indicated in the diagnosis field of the electronic medical record found on the database) and 9 954 976 visits were selected. These individuals were followed over time, and a total of 91 variables, including glycated hemoglobin (HbA1c), were checked periodically (on average every 0.6 years). The data preparation can be summarized as follows (for more in‐depth information please see^17^):

- Time interval between two HbA1c measurements ≥2 months.
- For each HbA1c measurement, “clinical factors” (eg, blood pressure, lipid panel, albuminuria, etc.) were tracked over time with an interval of maximum 4 months before and after the date of each measurement.
- For each HbA1c measurement, irreversible comorbidities (eg, acute myocardial infarction, stroke, etc.) were tracked starting from the date of first detection.

Table 1 provides the inclusion criteria for this study. Following the application of the inclusion criteria, measurements from 129 373 individuals were included.

**TABLE 1:** Inclusion criteria

Inertia‐yes	Inertia‐no
Patient not currently pregnant AND	Patient not currently pregnant AND
2 Patient currently on dual or triple therapy AND	2 Patient currently on dual or triple therapy AND
3 1–2 consecutive measurements of above threshold HbA1c >7.5% (58.5 mmol/mol) if patient is ≤75 years old; >8% (63.9 mmol/mol) if patient is >75 years old AND	3 1–2 consecutive measurements of above threshold HbA1c >7.5% (58.5 mmol/mol) if patient is ≤75 years old; >8% (63.9 mmol/mol) if patient is >75 years old AND
4 NO prescribed insulin therapy (basal, basal‐bolus or rapid) after second above threshold measurement	4 YES prescribed insulin therapy (basal, basal‐bolus or rapid) after either first or second above threshold measurement
The application of these criteria led to a total of 96 621 patients. Of this total 43 375 were started on insulin. The remaining 53 246 never received insulin	The application of these criteria led to a total of 32 752 patients

Abbreviation: HbA1c, glycated hemoglobin.

Data related to drug therapies and comorbidities were grouped as described in our previous study.^17^ Prescribed medications were grouped into eight main diabetes therapies to simplify the number of drug combinations, yielding 18 combinations. To ensure a robust estimate of comorbidities, we grouped information from across different fields in the electronic medical record.

**FIGURE 1:**
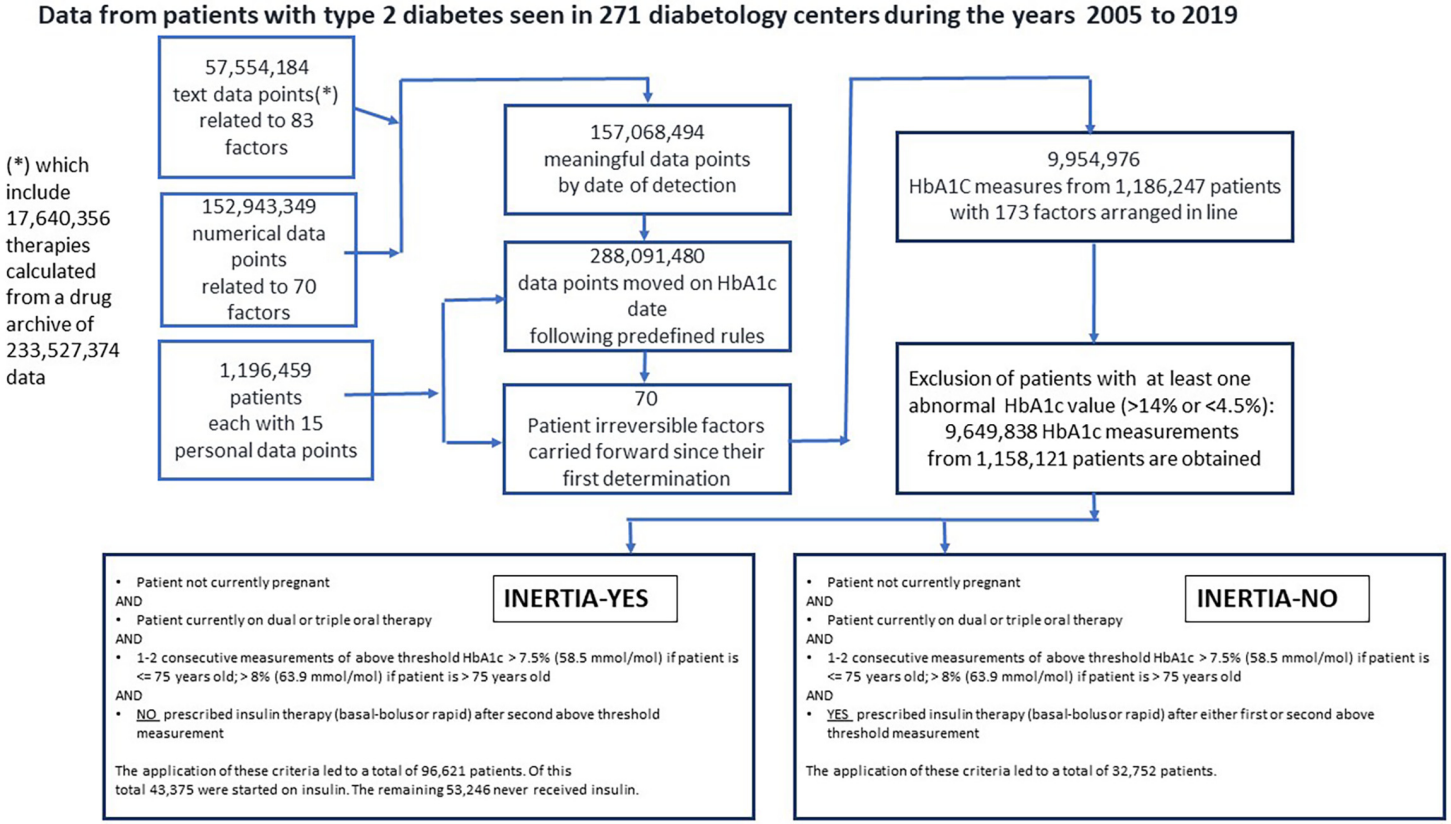
Flow chart illustrating the number of participants being included/excluded at each stage. HbA1C, glycated hemoglobin

Figure 1 provides a flow chart with participant characteristics.

Table 2 provides the means, median, SDs, and interquartile for the characteristics of the cohorts.

**TABLE 2:** Cohort characteristics

Cohort characteristics	INERT	NON‐INERT
BMI mean	30.5 kg/m^2^	29.9 kg/m^2^
BMI SD	5.6 kg/m^2^	5.9 kg/m^2^
BMI median	29.8 kg/m^2^	29.1 kg/m^2^
BMI (25 percentile)	26.7 kg/m^2^	25.8 kg/m^2^
BMI (75 percentile)	33.5 kg/m^2^	33.2 kg/m^2^
HbA1c mean	69 mmom/mol (8.5%)	79 mmom/mol (9.4%)
HbA1c SD	8.8 mmom/mol (0.8%)	14.3 mmom/mol (1.3%)
HbA1c median	67 mmom/mol (8.3%)	79 mmom/mol (9.1%)
HbA1c (25 percentile)	63 mmom/mol (7.9%)	68 mmom/mol (8.4%)
HbA1c (75 percentile)	73 mmom/mol (8.8%)	87 mmom/mol (10.1%)
Serum glutamic oxaloacetic transaminase (GOT) mean	23.1 mU/mL	25.1 mU/mL
GOT SD	13.4 mU/mL	27.8 mU/mL
GOT median	20.0 mU/mL	19.0 mU/mL
GOT (25 percentile)	16.0 mU/mL	15.0 mU/mL
GOT (75 percentile)	26.0 mU/mL	26.0 mU/mL
Serum glutamic pyruvic transaminase (GPT) mean	29.1 mU/mL	30.6 mU/mL
GPT SD	20.3 mU/mL	31.8 mU/mL
GPT median	24.0 mU/mL	23.0 mU/mL
GPT (25 percentile)	17.0 mU/mL	16.0 mU/mL
GPT (75 percentile)	34.0 mU/mL	34.0 mU/mL
High density lipoproteins (HDL) mean	47.7 mg/dL	46.6 mg/dL
HDL SD	13.1 mg/dL	14.0 mg/dL
HDL median	46.0 mg/dL	44.0 mg/dL
HDL (25 percentile)	39.0 mg/dL	37.0 mg/dL
HDL (75 percentile)	55.0 mg/dL	54.0 mg/dL
Low density lipoproteins (LDL) mean	99.5 mg/dL	99.4 mg/dL
LDL SD	34.8 mg/dL	37.5 mg/dL
LDL median	96.8 mg/dL	96.0 mg/dL
LDL (25 percentile)	76.0 mg/dL	73.8 mg/dL
LDL (75 percentile)	120.0 mg/dL	121.0 mg/dL
Systolic blood pressure (BP) mean	138.5 mmHg	137.7 mmHg
Systolic BP SD	18.3 mmHg	19.4 mmHg
Systolic BP median	140.0 mmHg	140.0 mmHg
Systolic BP (25 percentile)	128.0 mmHg	121.0 mmHg
Systolic BP (75 percentile)	150.0 mmHg	150.0 mmHg
Diastolic BP mean	79.2 mmHg	78.6 mmHg
Diastolic BP SD	9.6 mmHg	10.1 mmHg
Diastolic BP median	80.0 mmHg	80.0 mmHg
Diastolic (25 percentile)	70.0 mmHg	70.0 mmHg
Diastolic (75 percentile)	83.0 mmHg	82.0 mmHg
Triglyceride mean	158.2 mg/dL	177.5 mg/dL
Triglyceride SD	104.1 mg/dL	146.6 mg/dL
Triglyceride median	135.0 mg/dL	144.0 mg/dL
Triglyceride (25 percentile)	98.0 mg/dL	102.0 mg/dL
Triglyceride (75 percentile)	189.0 mg/dL	209.0 mg/dL
Fasting glycemia mean	171.5 mg/dL	207.6 mg/dL
Fasting glycemia SD	42.9 mg/dL	65.7 mg/dL
Fasting glycemia median	167.0 mg/dL	198.0 mg/dL
Fasting glycemia (25 percentile)	144.0 mg/dL	163.0 mg/dL
Fasting glycemia (75 percentile)	193.0 mg/dL	243.0 mg/dL
Glomerular filtration rate (GFR) mean	80.6 mL/min/1.73 m^2^	74.3 mL/min/1.73 m^2^
GFR SD	19.3 mL/min/1.73 m^2^	23.9 mL/min/1.73 m^2^
GFR median	83.9 mL/min/1.73 m^2^	77.6 mL/min/1.73 m^2^
GFR (25 percentile)	67.6 mL/min/1.73 m^2^	56.1 mL/min/1.73 m^2^
GFR (75 percentile)	94.5 mL/min/1.73 m^2^	93.1 mL/min/1.73 m^2^
Age mean	65.4 years	67.2 years
Age SD	9.8 years	11.3 years
Age median	66.0 years	68.0 years
Age (25 percentile)	59.0 years	59.0 years
Age (75 percentile)	72.0 years	76.0 years

### 2.2 LLM characteristics and ML modeling

ML has the ability to both analyze data without making any a priori assumptions and predict new output values from the data. The ML technique, “rule generation methods,” builds models described by a set of intelligible rules that permit the extraction of knowledge about variables in the analysis as well as their relationships with a target attribute. Two different paradigms for rule generation have been proposed in the literature. Decision trees^19^ adopt a divide‐and‐ conquer approach for generating the final model. Methods based on Boolean function reconstruction follow an aggregative procedure for building the set of rules.^20^, ^21^

LLM is a proprietary algorithm that implements the switching neural network model,^22^ which allows for solving classification problems and produces sets of intelligible rules expressed in the form: “if *premise* …, then *consequence* …,” where “*premise”* includes one or more conditions on the input variables, and “*consequence*” contains the output value or information about the target function in terms of “yes or no.” Thus, the LLM rule generation technique produces a subset of relevant variables associated with a specific outcome and informs on explicit intelligible conditions related to a particular outcome as well as relevant thresholds for each input variable. Furthermore, the “clear box” approach used by LLM yields “explainable AI,” which provides comprehensible and trustworthy results and output created by the ML algorithms.^23^

In the present study, data were subjected to a first stage of modeling to allow ML to select automatically the most relevant factors related to inertia. The model incorporated both descriptive variables (clinical and demographic) and dynamic variables (HbA1c gap and drop speed, mean, SD, and trend for several clinical measurements) collected from each individual (Table S1).

After the preliminary phase, four further modeling steps were completed to individuate the key variables that discriminate the presence or absence of inertia. The role and relevance of the different variables that influenced YES/NO inertia were taken through several modeling steps (learning set = 70% and test set = 30%) as outlined in Table 3.

**TABLE 3:** Description of model iterations

Step description	Input parameters from the AMD Annals database	Accuracy & AUC (area under the ROC curve)	Comment
Step 1 (first model iteration) All variables measured at the time of the patient visit (“static” clinical measurements from the patient and organizational measurements from the medical center), except for preceding HbA1c that was added later following LLM results and suggestions	Age, sex, body mass index (BMI), systolic blood pressure (BP), diastolic BP, HbA1c at current visit, fasting glucose, hypertension, dyslipidemia, triglycerides, High‐density lipoproteins (HDL), low‐density lipoproteins (LDL), creatinine, estimated glomerular filtration rate (eGFR), micro–macro/albuminuria, serum uric acid, nephropathy, atrial fibrillation, heart failure, stroke, cardiac complications, vasculopathy, lower limb complications, neuropathy, foot complications, eye complications, hepatopathy, serum glutamic oxaloacetic transaminase (GOT), serum glutamic pyruvic transaminase (GPT), drug therapy (double or triple), “years of clinical observation” (considered a proxy of duration of diabetes), Q‐score (quality of care summary score calculated for each year of observation, developed and validated in two previous studies)	Accuracy: 0.70 AUC: 0.760	This step in the model revealed a modest predictive precision (0.70), which indicated that the “static” variables were insufficient for the clear discrimination/forecast of the provider's decision (YES/NO inertia)
Step 2 (second model iteration) All variables measured at the time of the patient visit (“static” clinical measurements from the patient and organizational measurements from the medical center) as well as derived dynamic variables served as input for ML. In addition, HbA1c at previous visit was added based on Step 1 results and suggestions	Same as step1 + HbA1c at previous visit, HbA1c gap (HbA1c actual – HbA1c previous visit), HbA1c drop speed (speed of HbA1c yearly reduction) + mean, SD, and trend for HbA1c, fasting glycemia, systolic BP, diastolic BP, creatinine, eGFR, triglycerides, HDL, LDL, GOT, GPT, BMI	Accuracy: 0.79 AUC: 0.876	This model had very good predictive precision (0.79) and was >10% more precise than the first iteration. This second step clearly indicates that derived dynamic variables have a determining influential role on the provider's decision‐making process (YES/NO inertia)
Step 3 (third model iteration) For this iteration, only the variables linked to glycemia (static or dynamic) served as input for ML	HbA1c at current visit, HbA1c at previous visit + HbA1c drop speed, HbA1c gap, mean, SD, and trend for HbA1c and fasting glucose	Accuracy: 0.78 AUC: 0.845	This third iteration had decidedly good predictive precision (0.78), which is only slightly inferior to the second step in the model despite the fact that variables other than those related to glycemia were not included as input. This result indicates that glycemia and HbA1c play a dominant role over any other variable with respect to a provider's decision‐making process (YES/NO inertia)
Step 4 (Fourth model iteration) For this iteration all static and dynamic variables, excluding static and glycemic variables linked to glycemia (static or dynamic) (HbA1c and fasting glucose) served as input for ML	Same as first iteration (excluding HbA1c at current visit, fasting glucose + mean, SD, and trend for systolic BP, diastolic BP, creatinine, triglycerides, HDL, LDL, GOT, GPT, BMI, and eGFR)	Accuracy: 0.64 AUC: 0.676	This model confirms the dominant role of HbA1c and glycemia. This model, which excludes glycemic variables, has low predictive validity (0.64) relative to the first two. The variables used for input were not able to discriminate the outcome despite also being dynamic variables

Abbreviations: AMD, Italian Association of Medical Diabetologists; HbA1C, glycated hemoglobin; LLM, logic learning machine; ML, machine learning; ROC, receiver operating characteristics.

LLM affords the advantages of ML, which permits the analysis of very large number of variables, along with ability to have access to (transparency) of the ranking of the most relevant variables that can help guide the analysis.

As such, Step 1 began by incorporating all of the descriptive variables (Table S1) into the model, which resulted in accuracy = 0.70 and area under the curve (AUC) = 0.76 (also reported in Table S1). This was followed by Step 2 to verify if the addition of dynamically derived‐variables could improve the performance of the model. The hypothesis was that a medical practitioner's inertia could be influenced by factors related to the patient's progress across time rather than only by static parameters related to a single visit. This hypothesis was confirmed by the results from Step 2 in that the performance of the model significantly improved accuracy = 0.79 and AUC = 0.87. Furthermore, the relevant variables revealed by the transparent ML highlighted the important role of those variables that are related to the patient's progress. For example, the HbA1c gap achieved the second position in the ranking and in third position one finds the average HbA1c across 4 years (Table 3).

Given that the first three positions in the ranking of the variables in Step 2 were all related to HbA1c, we were driven to carry out a third step to verify if the dominant role of glycemia and HbA1c was real. Therefore, for Step 3, input for ML consisted only of variables that were related to glycemic factors (static and dynamic values for fasting HbA1c and glycemia). Step 3 results had accuracy = 0.78 and AUC = 0.84, which confirm the dominant role of the glycemic factors as determinants of the medical practitioner's decision to initiate insulin therapy in a patient.

As a counterproof to Step 3, we carried out a final step in modeling that included the use of all variables, both dynamic and descriptive, EXCEPT for those related to fasting HbA1c and glycemia, as input for ML. The results from Step 4 of modeling resulted in accuracy = 0.64 and AUC = 0.67. These results were modest compared to the previous three modeling steps and serve to demonstrate the small, but not absent, influence that other variables have on the medical practitioner's decision to initiate insulin therapy.

## 3 RESULTS

The total data pool was comprised of 129 373 individuals, 32 752 of whom were started on insulin therapy in accordance with the 2020 guidelines of the American Diabetes Association, 43 375 whose insulin therapy initiation was delayed, and 53 246 who never received insulin as reported in Table 1.

The results indicate that the best model was derived from the second modeling iteration. This best performing model, which includes all the dynamic and descriptive variable outlined in Table S1, underscores the relevance of the variables selected for the analysis and prediction of the phenomenon studied. The area under the receiver operating characteristic curve (ROC) of the model is = 0.87 (Figure 3), accuracy = 0.79, sensitivity = 0.76, specificity = 0.78, and precision = 0.91. The 10 main variables in terms of relevance highlight the dominant role of current HbA1c values as well as the immediately preceding values.

Table 4 provides a ranking of the most relevant factors in the ML model. The factors with the highest relevance, namely those having the strongest correlation with outcome inertia‐yes/inertia‐no, are the first three listed and are related to consecutive HbA1c measurements and the measured difference between them, or HbA1c gap. Average HbA1c across 4 years, fasting glucose, as well as yearly HbA1c reduction speed are also relevant, but to a lesser degree than the HbA1c gap.

**TABLE 4:** Ranking of most relevant factors for patients experiencing or not experiencing insulin therapy inertia

Relevant factors	Threshold inertia‐no	Threshold inertia‐yes	Relevance
Factors related to glycemia			
HbA1c at current visit	>73 mmol/mol (>8.8%)	<72 mmol/mol (<8.7%)	0.741
Change in HbA1c from previous visit (HbA1c gap)	>11 mmol/mol (>1.0%)	<6.6 mmol/mol (< 0.6%)	0.396
Mean HbA1c (4 previous years)	>69 mmol/mol (>8.5%)	< 66 mmol/mol (<8.2%)	0.199
Fasting glucose	>242	<210	0.186
HbA1c reduction speed (a negative drop in speed indicates an annual worsening of HbA1c relative to the indicated threshold)	<−11 mmol/mol (<−1.0%)	>−5.94 mmol/mol (>−0.54%)	0.158
Mean fasting glucose (4 previous years)		<203	0.026
Fasting glucose trend		<1.33	0.025
HbA1c trend		<0.05	0.013
Factors related to insulin resistance			
Mean diastolic BP (4 previous years)	< 88		0.029
BMI		>24	0.028
Diastolic BP trend		>−0.53 & <+0.15	0.020
Triglyceride trend		>−1.97 & <+2.34	0.017
Average BMI (4 previous years)		>24	0.010
Comorbidity			
Mean eGFR (4 previous years)	< 59.99	>69	0.044
Hepatopathy (threshold)		Not diagnosed	0.040
Nephropathy		Not diagnosed (OR current dialysis)	0.039
Hyperuricemia		Not diagnosed	0.028
Average creatinine (4 previous years)	>1.09	<0.75	0.026
eGFR trend		>−0.99 & <+0.01	0.026
Heart disease	Diagnosed	Not diagnosed	0.019
eGFR	<77		0.016
Personal characteristics			
Age	>78	<64	0.155
History and care			
Months of follow‐up	>101		0.111
Q‐score		<21	0.048

Abbreviations: BMI, body mass index; BP, blood pressure; eGFR, estimated glomerular filtration rate; HbA1C, glycated hemoglobin.

The model indicates that the glycemic profile, which for the purposes of this study refers specifically to the change in either HbA1c or glycemia seen in a patient from one visit to the next, and a patient's glycemic trend, that is, the direction that change takes, has a greater effect on a provider's therapeutic inertia than any one individual datapoint in the patient's static profile. ML indicates that for threshold HbA1c values, on average, an HbA1c gap of <6.6 mmol/mol (<0.6%) is correlated with inertia. On the other hand, an HbA1c gap of >11 mmol/mol (>1.0%) is correlated with non‐inertia. Thus, the data suggest that the HbA1c gap between two consecutive visits appears to play a crucial role in the decision to start insulin therapy in a person with type 2 diabetes. Moreover, not only are the current HbA1c and the change in HbA1c between two consecutive measurements the key drivers with the strongest influence on the presence or absence of inertia, but the average HbA1c across 4 years is also relevant in the decision‐making process. Furthermore, in terms of relevance, following the HbA1c level at the time of the most recent visit, ML suggests that below a value of 72 mmol/mol (8.7%), clinical inertia is most probable, whereas values above 73 mmol/mol (8.8%) lead to a greater probability that insulin will be initiated.

There are other parameters not directly linked to HbA1c but which in all iterations of modeling also suggest some relevance. For example, estimated glomerular filtration rate (eGFR) (mean and trend) stood out in all three iterations of modeling as the most important comorbidity and, when the eGFR mean is <59.99, it is correlated with non‐inertia. A body mass index >24, either as a static variable or as the mean over 4 years, is correlated with inertia. Both stable triglycerides and the absence of complications in particular cardiac, hepatopathy, and hyperuricemia are all correlated with a greater probability of inertia.

The model was also able to confirm data previously reported in the literature. Namely, HbA1c values of less than 68 mmol/mol (8.4%) are associated with insulin therapy inertia whereas values above 75 mmol/mol (9.0%) are associated with an increased probability of insulin therapy initiation. The presence of a sudden increase in HbA1c as a driver of non‐inertia led to a more in‐depth analysis to verify the accuracy of the results provided by LLM.

Given that among the transparent ML rankings the HbA1c gap is an innovative finding in that it has not yet been described in the literature as a factor that plays a role in insulin inertia, we wanted to verify with traditional statistics the correlation between the variables revealed by transparent ML and insulin inertia. To confirm the results from the model, a statistical analysis was carried out on those individuals identified by ML who at some point in their clinical history had HbA1c values of >58 mmol/mol (>7.5%) for one or two consecutive visits. HbA1c was compared between inertia‐YES and inertia‐NO conditions at "T0." In the inertia‐YES condition, T0 represents the point at which an individual presents with an HbA1c >58 mmol/mol (>7.5%) for the second consecutive time and is the point at which insulin therapy initiation would have been appropriate.^24^ In the inertia‐NO condition, T0 represents the point at which a patient who has had an HbA1c >58 mmol/mol (>7.5%) for one or two consecutive visits is prescribed insulin. The total data pool comprised 129 373 individuals, 32 752 of whom were started on insulin therapy in accordance with the 2020 guidelines of the American Diabetes Association, 43 375 whose insulin therapy initiation was delayed, and 53 246 who never received insulin as reported in Table 1.

**FIGURE 2:**
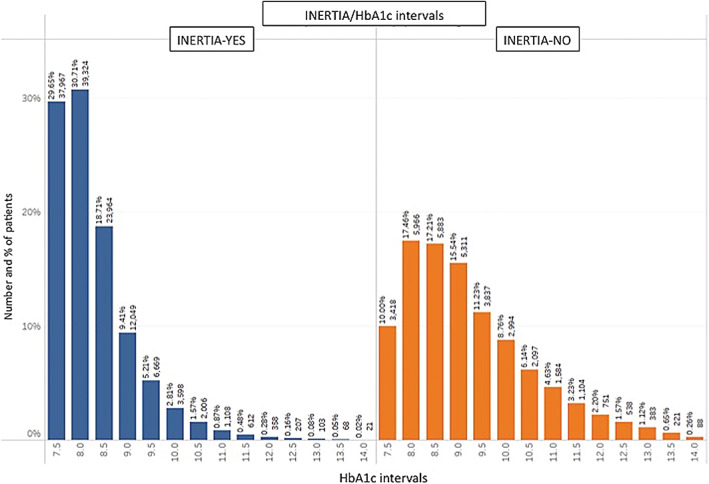
Stratification of HbA1c levels at "T0" across patients who experienced a delay in insulin initiation (Inertia‐yes) and those who did not (Inertia‐no)

Figure 2 provides an illustration of the stratification of HbA1c levels across patients who experienced a delay in insulin initiation (inertia‐yes) and those who did not (inertia‐no).

**FIGURE 3:**
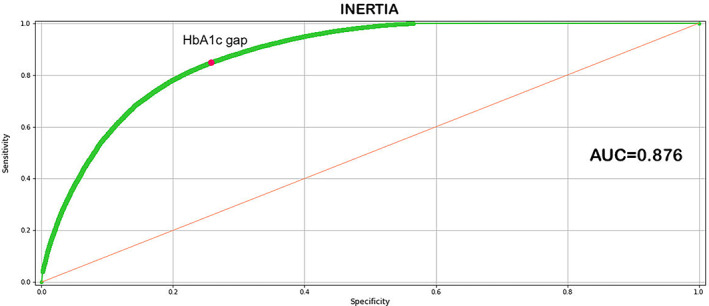
Area under the receiver operating characteristic (AUC ROC) curve of the best performing model

**FIGURE 4:**
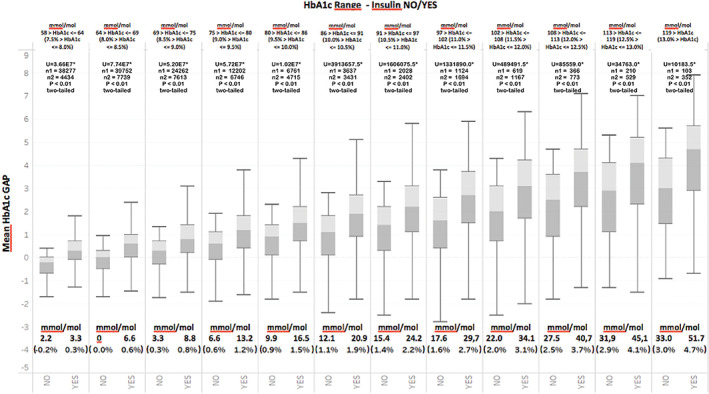
Average HbA1c gap across HbA1c ranges at T0 for individuals who did and did not experience therapeutic inertia. Mann–Whitney (*U*) and number of subjects is given for each range. (*) indicates statistical significance

**FIGURE 5:**
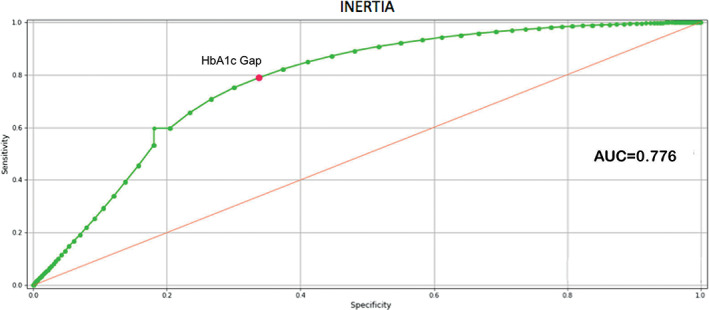
Area under the receiver operating characteristic (AUC ROC) Curve of the HbA1c gap relative to YES/NO inertia

**FIGURE 6:**
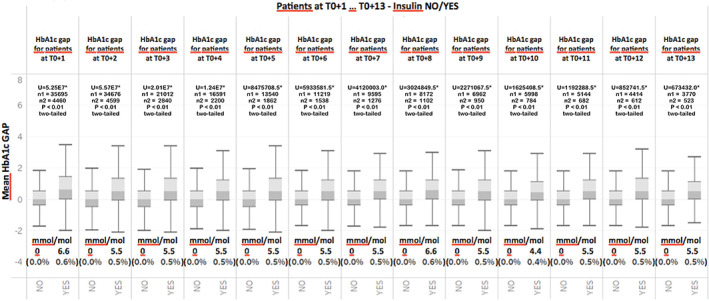
HbA1c variation for subgroups of individuals who are initiated on insulin either at T0 + 1 or on subsequent visits (T0 + 2…T0 + n). Mann–Whitney (*U*) and number of subjects is given for each range. (*) indicates statistical significance

The primary goal of the statistical analyses was to verify if the HbA1c gap was able to discriminate the presence or absence of inertia for any HbA1c level at a particular visit. Figure 4 shows the calculated HbA1c gap across different ranges of HbA1c at T0 for individuals who did and did not experience therapeutic inertia. What can be observed in Figure 4 is that the HbA1c gap is systematically higher across all ranges of HbA1c in those individuals who did not experience therapeutic inertia. To confirm the statistical validity of the results, we began by determining whether the data have a normal distribution by submitting the entire range of HbA1c values to a Jarque–Bera test, which is typically used for large data sets such as ours. The results of the test indicated a nonnormal distribution (*p* <.05). The data set were then submitted to a Mann–Whitney test for each of the pairwise comparisons starting with 58–64 mmol/mol (7.5%–8%) and ending with 119 mmol/mol (13%). The analyses confirmed a statistically significant difference in the average HbA1c gap between the two groups (presence of absence of inertia) (*p* <.01, two tailed test) across all ranges.

A further verification was made by calculating the ROC curve for the HbA1c gap related to inertia. Figure 5 shows the AUC (0.776) that confirms the ability of the HbA1c gap to discriminate between situations of therapeutic inertia from those where no therapeutic inertia was present as suggested by ML. The Youden index for the HbA1c gap indicates a threshold value equal to 5.5 mmol/mol (0.505%), which agrees with the threshold value obtained with ML. It should be noted that the threshold values obtained with the two techniques were not identical, given that ML takes into consideration the HbA1c gap along with all other variables input into the model, and the Youden index refers strictly to the HbA1c as a single variable.

Finally, in order to verify if the HbA1c gap plays a role in the discrimination between situations in which therapeutic inertia is present and those situations in which it is not even after T0, all patient visits after T0 (T0 + 1, T0 + 2…T0 + n) were analyzed and the HbA1c gap was calculated. Figure 6 illustrates the results of this analysis for individuals who received insulin and those who continued to experience therapeutic inertia. Figure 6 once again shows that even for patient visits subsequent to T0, the HbA1c gap is systematically elevated in those individuals who receive insulin relative to those who continue to experience therapeutic inertia. The Jarque–Bera test was also applied to this series of data and results indicated a nonnormal distribution (*p* < .05). A Mann–Whitney test revealed that for all periods following T0, average values between the two groups were statistically significant (*p* < .01, two‐tailed test). This finding further confirms the discriminating role of HbA1c between situations in which therapeutic inertia is present and those in which it is not.

## 4 DISCUSSION

The current state of therapeutic inertia and delay in initiation of insulin therapy in individuals with type 2 diabetes is systemic and unsettling.^2^, ^3^ This inadequate approach to glycemic control in individuals with type 2 diabetes is a global concern and one that is related to poor patient outcome as well as increased socioeconomic and clinical burden.^4^, ^5^, ^25^, ^26^ Though several studies have provided explanations for the motivation behind the delay in insulin therapy,^27^, ^28^ clearly, new approaches are needed to get at the root of the factors that are associated with therapeutic inertia, particularly the identification of key drivers that may break a health care provider's tendency to delay initiation of therapy.

Our objective for this study was to uncover as yet unrecognized factors that motivate a provider to move away from behavioral inertia and initiate insulin therapy using ML techniques and evidence‐based medicine. LLM, a “clear box” ML with “explainable AI,” is able to delineate the characteristics of those individuals who face therapy initiation inertia compared with those who undergo treatment with insulin in accordance with established guidelines. It was thus possible to establish a predictive model capable of identifying key drivers associated with the initiation of insulin therapy with high accuracy (0.79).

Specifically, our results revealed that a medical practitioner was more likely to initiate insulin not only as a consequence of excessively elevated HbA1c as one might expect, but also when, across two consecutive visits, a patient showed a difference in HbA1c of 1% or greater. This difference, which we are calling the HbA1c gap, is correlated with a movement toward insulin initiation irrespective of the absolute HbA1c value and could play an important role in the decision to start insulin. That is to say, it was the HbA1c gap that prompted insulin initiation rather than only the absolute HbA1c at a current visit, which in our study varied between 7.5% and 11%.

We believe that our results could provide the medical practitioner with an additional and new measure to monitor in existing or new patients. One could even envision the inclusion of an AI algorithm as part of a patient's electronic health record, which could alert the provider in real time as to risks related not only to inertia but to other health concerns that can be modeled in a manner similar to what has been described in this report. This type of analysis which has been deemed “augmented intelligence” is a way to use AI to improve the quality of decision‐making rather than substitute or automate human decision‐making. Rather than waiting for a critical point at which the patient begins to experience new and potentially dangerous comorbidities as the moment to begin insulin therapy, the medical practitioner could remain increasingly more aware of variabilities in a patient's condition in real time and intervene more promptly and appropriately.

The model's high level of precision confirms the adequacy and utility of the input variables to identify key drivers that influence a health care provider's tendency to exhibit or not exhibit inertia when faced with an individual with type 2 diabetes. The model clearly points to the role of dynamic variables related to glycemia as crucial for the determination of whether or not a health care provider will make a timely decision or remain inert with respect to the initiation of insulin therapy.

A weakness in the study is related to the need to validate findings in a pilot study first. Furthermore, a weakness of any ML model is its reliance on electronic medical data, which to be fully functional for use with ML, must somehow remain in a public domain and not be privately owned data. Otherwise, access to the data can be terminated when an attempt is made at integration with ML modeling from external software.

The data from this study provide new insight for health care providers as they face the challenges of understanding their patients and provide the individualized care that each needs. The information generated by these LLM analyses not only will allow health care providers to gain awareness into the factors that drive their behavior leading to therapeutic inertia but also clearly paves the way for in‐depth investigations of other unknown factors using ML techniques that could help identify further subgroups at greater risk for therapeutic inertia.

In sum, the presence of inertia in the absence of complications suggests how the importance of timely and decisive therapeutic action is still often underestimated even though current guidelines clearly indicate that timely therapeutic action prevents future complications. The present real‐life study has demonstrated how research on inertia can generate new points of view and novel approaches, which will provide healthcare providers the ability to create innovative, effective, and realistic training on this highly debated topic.

## DISCLOSURE

The authors declare that there is no conflict of interest. This study used existing data located on a database and as such did not involve the direct participation of humans or animals. Therefore, no ethics board approval was required. Furthermore, no data used in this study had any identifiable features that could be traced back to the individual from whom the data were obtained.

## Supporting information


**Table S1.** Variables collected from each patient
