# Supplementary material for: Transparent machine learning suggests a key driver in the decision to start insulin therapy in individuals with type 2 diabetes
Source: J Diabetes. 2023 Mar 8;15(3):224–36. doi: 10.1111/1753-0407.13361 (PMC10036260; doi:10.1111/1753-0407.13361)
Supplement: Supplementary file 1 — Table S1. Variables collected from each patient [file JDB-15-224-s001.docx]

**Supplemental Table 1** Variables collected from each patient

| **Type of Variable** | **List of variables included in each type** |
| --- | --- |
| Descriptive | Age, Sex, Body Mass Index (BMI), Systolic Blood Pressure (BP), Diastolic BP, HbA1c at current visit, HbA1c at previous visit, Fasting glucose, Hypertension, Dyslipidemia, Triglycerides, High Density Lipids (HDL), Low Density Lipids (LDL), Creatinine, Estimated Glomerular Filtration Rate (eGFR), Micro/Macro albuminuria, Serum uric acid, Nephropathy, Atrial fibrillation, Heart failure, Stroke, Cardiac complications, Vasculopathy, Lower limb complications, Neuropathy, Foot complications, Eye complications, serum glutamic oxaloacetic transaminase (GOT), serum glutamic pyruvic transaminase (GPT), Hepatopathy, Drug therapy (double or triple), “years of clinical observation” [considered a proxy of duration of diabetes], Q-Score [quality of care summary score calculated for each year of observation, developed and validated in two previous studies] (29, 30) |
| Dynamic-derived variables | HbA1c gap (HbA1c actual – HbA1c previous visit), HbA1c drop speed [speed of HbA1c yearly reduction - if HbA1c has increased, this is a negative number].  Mean, standard deviation, and trend (for the last 4 years when available, otherwise for the available number of years) |
